# Comparing mixed oil to soybean oil lipid emulsion in patients on home parenteral nutrition: a pilot prospective double-blind, crossover, randomized trial

**DOI:** 10.1186/s40814-023-01295-1

**Published:** 2023-04-20

**Authors:** Nayima M. Clermont-Dejean, Katherine J. P. Schwenger, Celeste Arca, Nicha Somlaw, Amnah Alhanaee, Taís Daiene Russo Hortencio, Jennifer Jin, Hyejung Jung, Wendy Lou, David Ma, Johane P. Allard

**Affiliations:** 1 Department of Medicine, Toronto General Hospital, University of Toronto, 585 University Avenue, 9N-973, Toronto, ON M5G 2N2 Canada; 2 Department of Medicine, Chulalongkorn University and King Chulalongkorn Memorial Hospital, The Thai Red Cross Society, Bangkok, Thailand; 3 Tawam Hospital, Abu Dhabi Health Authority, Abu Dhabi, United Arab Emirates; 4 State University of Campinas, Unicamp, São Paulo, Brazil; 5 Sao Leopoldo Mandic Institute and Research Center, Campinas, São Paulo Brazil; 6 Division of Gastroenterology, Department of Medicine, University of Alberta, Edmonton, AB Canada; 7 Dalla Lana Public Health Department, University of Toronto, Toronto, ON Canada; 8 Department of Human Health and Nutritional Sciences, University of Guelph, Guelph, Canada

**Keywords:** Home parenteral nutrition, Mixed oil lipid emulsion, Soybean oil lipid emulsion, Liver function

## Abstract

**Background:**

Home parenteral nutrition (HPN) can be associated with increased liver enzymes, catheter-related bloodstream infections (CRBSI), and hospitalizations. Mixed oil (MO) versus soybean oil (SO) lipid emulsion reduces risks in hospitalized patients, but there are no randomized double-blinded controlled trials in HPN. Therefore, the primary objective was to test the study’s feasibility such as recruitment and retention in the HPN population and the secondary objective was to assess changes in liver enzymes between MO and SO as well as other clinical and biochemical outcomes.

**Methods:**

This 13-month prospective double-blind crossover randomized pilot trial took place in Toronto, Canada. Participants were HPN patients who were a part of the HPN program at Toronto General Hospital. We recruited patients from the HPN program. HPN patients receiving SO were randomized to either MO or SO, and the study duration was 6 months in each arm (MO or SO) with a 1-month washout period resuming SO. As this is a crossover trial design, the patient is his/her own control. The main outcome measures were descriptions of study feasibility, namely the study recruitment and retention. We also collected biochemical parameters, CRSBI, hospitalization rate, antibiotic use, and mortality. Demographic, nutritional, clinical, and laboratory data were collected at baseline, 3 and 6 months of each arm. The primary analysis population was defined as the per-protocol population who completed the trial including all lipid measurements.

**Results:**

A total of 65 HPN patients were assessed, and 60 met the inclusion criteria for the study. Thirty-five percent (21/60) were randomized using a computer-generated random number sequence generator: 10 participants were randomized to receive SO first while 11 were randomized to receive MO first. At 13 months, 3/10 who received SO first completed the study, whereas 9/11 who received MO first completed the study. This did not meet our a priori criteria for success in recruitment and retention. Between types of lipid emulsions, there were no significant differences in changes in liver enzymes or biochemical and clinical outcomes, despite significant changes in plasma free fatty acid composition reflecting MO or SO.

**Conclusions:**

Overall, this pilot trial demonstrated that the use of a prospective double-blind, crossover, randomized trial design was not feasible to conduct in the HPN population because of difficulties in recruiting and retaining patients. In addition, there was no significant impact of MO versus SO lipid emulsion on liver enzymes or most parameters. The lack of significance may be attributed to low sample size from low recruitment and high drop-out rate, short study duration (6 months/arm), and complex care. In a future definitive trial, a multicenter study of longer duration and a larger sample size is recommended, and drop-outs may be reduced by using a parallel study design.

**Trial registration:**

ClinicalTrials.gov, NCT02796833. Registered on 13 June 2016—retrospectively registered.

**Supplementary Information:**

The online version contains supplementary material available at 10.1186/s40814-023-01295-1.

## Key messages regarding feasibility

- What uncertainties existed regarding the feasibility?
  a Participant recruitment, enrollment, and participation—could we reach the desired sample size?
  b Adverse events and drop-out rate with a cross-over study design in this patient population.
  c Study duration—would 6 months be enough time to detect significant differences in primary outcomes between the two lipids?
- What are the key feasibility findings?
  a The sample size was not feasible given the limited number of willing participants as well as the high drop-out rate.
  b The inclusion of only one center did not allow for enough people to be recruited and enrolled in this study.
  c The crossover study design was not ideal in this patient population as many people did not complete the study and thus impacted the significance of the findings
- What are the implications of the feasibility findings for the design of the main study?
  a A multicenter study of longer duration and larger sample size is recommended. A parallel design may be preferable.

## Introduction

Home parenteral nutrition (HPN) is provided to patients with chronic intestinal failure (IF) from various causes who are not able to sufficiently absorb fluids and nutrients to meet nutritional requirements and ensure survival [1]. Parenteral nutrition (PN) includes macronutrients in the form of dextrose, amino acids, and lipid emulsions containing triglycerides and phospholipids, in addition to electrolytes, trace elements, vitamins, and water [1]. Intravenous lipid emulsion is the major source of energy and essential fatty acids and can be based on various oils [1]. Among them, soybean oil (SO) was the first commercially available lipid emulsion; however, over the years, studies have shown that its long-term use may have a negative impact on the inflammatory and immune responses due to the high (ω-6) polyunsaturated fatty acid (PUFA) content [2–6]. More recently, new lipid emulsions have reduced (ω-6) PUFA content to alleviate these potentially detrimental effects [7]. One of these newer commercial products used a mixture of oils that contain fish oil-derived long-chain (ω-3) PUFAs that have been shown to reduce inflammation, improve microcirculation, and reduce PN-associated liver disease [2, 4, 6, 8–10]. Two decades ago, a mixed oil (MO) lipid emulsion containing 30% soil oil, 30% medium-chain triglycerides, 25% olive oil, and 15% fish oil was introduced. This MO lipid emulsion was found to be safe and well tolerated in both adult and pediatric populations [2, 5, 6, 8, 10–18]. It was also associated with a shorter length of hospital stay [16], reduction in rates of nosocomial infection [19, 20], and improvement of liver enzyme profile and PN-related liver disease [8, 17, 21]. Although there is a lack of data from larger long-term randomized controlled trials within the HPN population, published clinical data does highlight some benefits of using MO in patients with type I and II IF [11–13, 15–20, 22], with only a few studies concentrating exclusively on the chronic type III intestinal failure patients receiving HPN [14, 23]. Type I is defined as an acute, short-term, and usually self-limiting condition, and type II is characterized by prolonged acute condition, often in metabolically unstable patients, requiring complex multidisciplinary care and intravenous supplementation over periods of weeks or months [24]. Few studies have focused exclusively on chronic type III IF in patients receiving HPN [14, 23] where type III IF is defined as a chronic condition, in metabolically stable patients, requiring intravenous supplementation over months or years [24]. These studies evaluated the impact of MO when used from 2 months [23] up to 5 years [25]. One was a cohort study of 17 patients followed for at least 12 months; results found that MO was well tolerated in those with SO lipid intolerance and MO allowed for improvement of the macronutrient composition in the PN with a decrease in dextrose energy and increase in lipid energy [14]. It also showed improvement in alanine transaminase (ALT), aspartate transaminase (AST), and total bilirubin levels [14]. Another study with a prospective design compared two parallel groups, MO (*n* = 13) and olive oil (OO) based (*n* = 19) PN over a 60-day period [23]. The results showed that both MO and OO-based lipid emulsions significantly altered the fatty acid profile and that MO did not alter liver function markers of inflammation; however, OO significantly decreased gamma-glutamyl transpeptidase (GGT) and interleukin-8 [23]. Finally, an open-label study reported on 65 subjects randomized to receive one of three lipid emulsions (MO vs SO vs OO) over a 5-year period [25]. No difference in liver enzymes or bilirubin was found, and the rate of catheter infection between types of lipid emulsion was not significantly different. No other clinical outcomes were assessed [25].

Due to a lack of data on the HPN patient population, we recently performed a 2-year prospective cohort study with 120 subjects (MO:68; SO:52) comparing MO to SO lipid emulsion using the Canadian HPN Registry [26]. Both groups were similar at baseline except for, in MO, a higher use of the Hickman line (62.12 vs 42%, *p* = 0.038) and more western Canada-based hospital care (75 vs 42.31%, *p* < 0.0002) [26]. We found that the MO group had significantly more hospitalizations (*p* < 0.001), more hospitalizations related to HPN (*p* < 0.012), and more hospitalization days related to HPN (*p* < 0.016) per patient per year compared to SO patients [26]. There was no significant difference between groups for line sepsis per 1000 catheter days (MO 0.05 (0.0, 1.0) vs SO 0.0 (0.0, 0.22), *p* = 0.053) or mortality [26]. Overall, all other variables, including biochemical, were similar between groups with no significant differences in changes [26]. In a multiple regression analysis, the following factors were significantly associated with a greater number of hospitalizations per patient per year: use of MO, high blood glucose from the last recorded value, and having died by the end of the study period [26]. Therefore, from this study, results suggest an increased risk of hospitalization in HPN patients receiving MO lipid emulsion versus SO. These findings conflict with previous reports showing clinical advantages associated with the use of MO in studies of shorter duration with smaller sample sizes. To further assess the long-term effect of using MO lipid emulsion in HPN patients, a large randomized controlled trial should be performed. In preparation for this and in parallel to the cohort study, we conducted the present pilot double-blind randomized controlled trial to determine the sample size and feasibility of such a trial using the crossover design. The cross-over design was used primarily because of the small and complex HPN patient population which makes recruitment challenging. In addition, there is a large heterogeneity between patients due to the various causes of IF and gastrointestinal anatomy that are associated with a wide spectrum of oral intake and absorptive capacity leading to various HPN regimens. In this study, the primary objective was to examine the feasibility of recruitment and retention of patients on HPN. Secondarily, the objectives of this study were to compare MO to SO lipid emulsion with the outcome being liver enzymes. Other outcomes of interest were also monitored and included other laboratory parameters, number of hospitalizations, number of catheter-related bloodstream infection (CRBSI), antibiotic use, and mortality.

## Materials and methods

### Design

This was a prospective, single-center, double-blind, two-armed, crossover randomized clinical trial of a 13-month duration aimed to demonstrate that long-term use of MO lipids is better for liver function compared to SO lipids in HPN patients. This study was approved by the University Health Network Research Ethics Board (REB#14–8537) and was listed on ClinicalTrials.gov (NCT02796833). Adults who were on a stable regimen of SO and HPN for at least 6 months were expected to require long-term HPN for at least 13 months and were clinically stable for at least 4 weeks with no acute medical comorbidities were included in this study. Exclusion criteria included subjects who were not already on SO HPN, those requiring short-term (less than 13 months) PN supplementation, those with the inability to provide informed consent, those with alcohol or drug abuse, those who were pregnant or lactating, and those in a clinically unstable condition.

### Sample size

Because this was a pilot study to assess feasibility and sample size for a future randomized controlled trial, no sample size was calculated, and all patients in our HPN programs that could travel for study visits and who met the study criteria were enrolled. Our HPN program includes 60–65 HPN patients and covers the province of Ontario, Canada. The number of patients in the present pilot study was similar to that in the few previous studies that used different designs for comparing MO to SO, in which 17–73 subjects were assessed [11, 14, 23, 25].

### Randomization and blinding

Randomization was performed using www.randomizer.org, a computer-generated random number sequence generator, and the allocation was concealed using a sequentially numbered table kept by the pharmacy. The lipid emulsion assignment was not known (i.e., blinded) to the nutrition support team and patients, except for a designated PN pharmacist who was not participating in the study. The biostatisticians were blinded to the study as well.

### Recruitment and intervention sequence

**Fig. 1:**
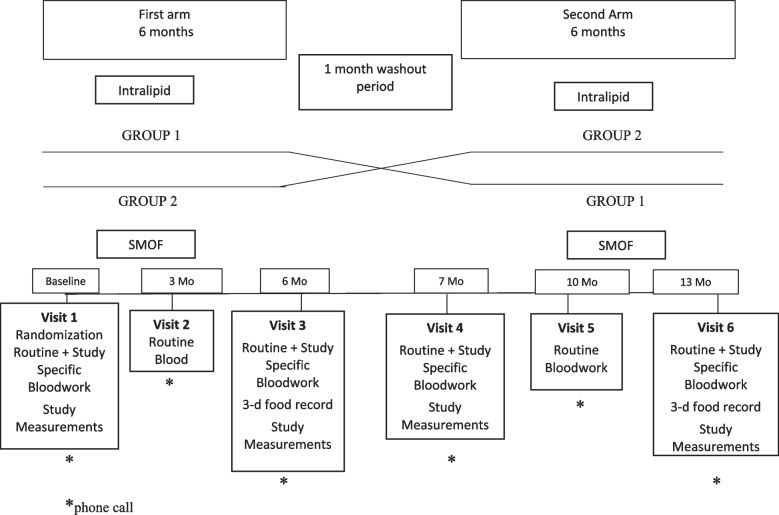
Trial protocol

Subjects were recruited by a registered nurse from the HPN clinic at the University Health Network, Toronto, Ontario. During a routine clinical checkup, patients were recruited for the study and provided a consent form. After providing informed consent, patients were enrolled and randomized into two groups scheduled to participate in the two arms of the study, lasting 6 months each, separated by a 1-month washout period during which both groups received SO. SO was chosen as the washout lipid as it is the primary lipid used in this HPN program. During the study, each patient participated in follow-up appointments at the start, middle, and end of each study arm for a total of six visits. Randomization was performed during the patient’s first visit. Visits at the start and end of each study arm included routine and study-specific bloodwork as well as study measurements (visits 1, 3, 4, and 6). At the end of each arm, the patient submitted a three-day food record. The blood for routine bloodwork was drawn from each patient during the middle of each study arm (visits 2 and 5). Patients were seen in person/telehealth at visits 1, 3, 4, and 6 and patients received a phone call prior to each visit (Fig. 1). As this is a crossover trial design, the patient is his/her own control.

### Variables and their measures

#### Primary feasibility outcomes

The primary feasibility outcomes were recruitment and retention rate. The recruitment rate was calculated as the percentage of HPN patients who consented to be in the study among those who were approached. The retention rate was defined as the percentage of HPN patients who remained until the end of the study among those who were enrolled at baseline.

#### Secondary patient-centered outcomes

Based on previous studies [8, 17], the primary outcome was to evaluate the changes in mean ALT values when using MO compared to SO. Secondary outcomes included additional biochemical tests such as liver function tests (AST, ALP, GGT, total bilirubin, conjugated bilirubin, and albumin), coagulation markers (aPTT, INR, and CRP), and general biochemistry (hemoglobin, WBC count, platelets count, sodium, potassium, bicarbonate, chlorine, phosphate, calcium, and magnesium), evaluated for both lipids. Lipid profile, total cholesterol, triglyceride levels, and fatty acid levels (linoleic acid, alpha-linolenic acid, eicosapentaenoic acid [EPA], docosahexaenoic acid [DHA], ω-6 to ω-3 ratio, and arachidonic acid) were also evaluated. Monitored clinical outcomes included new catheter-related bloodstream infection (CRBSI), new infections including CRBSI, change of vascular access, use of antibiotics, duration of antibiotic therapy (days), number of unexpected hospitalizations, surgery, death, adverse events, and serious adverse events including serious adverse events probably related to the intervention. Study dropouts were tracked for both MO and SO treatment groups.

### Laboratory measurements

Blood samples for study purposes were collected at visits 1, 3, 4, and 6 and analyzed by routine methods at the accredited hospital laboratory (Laboratory Medicine Program, University Health Network). This included albumin (g/L), total bilirubin (μmol/L), conjugated bilirubin (μmol/L), liver enzymes (alkaline phosphatase [ALP], alanine transaminase [ALT], aspartate aminotransferase [AST], and gamma-glutamyl transferase [GGT]; [U/L]), total cholesterol (mmol/L), triglycerides (mmol/L), activated partial thromboplastin time (aPTT) (s), international normalized ratio (INR), C-reactive protein (CRP) (mg/L), hemoglobin (g/L), white blood cells (WBC) (× 10^9^/L), platelets (× 10^9^/L), sodium (mmol/L), potassium (mmol/L), bicarbonate (mmol/L), phosphate (mmol/L), calcium (mmol/L), and magnesium (mmol/L). For plasma PUFA measurements, blood samples were collected in EDTA-containing tubes and immediately centrifuged. The plasma was collected and subsequently frozen at − 80 °C as previously described [27, 28]. Lipids were extracted from plasma using the Folch method [27].

### Statistical analysis

Due to the considerable number of discontinuations, the primary analysis population was defined as the per-protocol population who completed the trial including all lipid measurements taken at the beginning and end of both trial periods. Demographic data and baseline characteristics were displayed separately by sequence group, e.g., SO first followed by MO, and vice versa. Continuous variables were summarized using means and standard deviations (SDs), and categorical variables were expressed as counts and percentages, with the difference in the means (95% CI) and difference in the proportions (95% CI) between sequence groups estimated using the t-distribution and Chan and Zhang’s exact test, respectively.

The difference (95% CI) of the changes in the mean ALT level, and other continuous clinical outcomes were estimated between SO and MO treatment groups using analysis of covariance (ANCOVA) models for repeated measures with treatment period, type, and sequence as fixed effects and subject as a random effect. The risk difference for binary clinical outcomes and safety data were estimated using generalized linear model regression with binomial distribution and logit link.

The heterogeneity of the treatment effect across the values of each lipid enzyme was explored with respect to ω-6, ω-3, EPA, and DHA using ANCOVA models for repeated measures. The models incorporated fixed effects of treatment, lipid enzyme, and treatment by lipid enzyme interaction, and a random effect of the subject. Due to the small sample size, we did not present *p* values for comparisons. Given the small sample size, the results obtained should be considered preliminary and interpreted with caution. All statistical analyses were performed using SAS version 9.4 (SAS Institute Inc., Cary, NC, USA).

## Results

### Patient population and primary feasibility outcomes

**Fig. 2:**
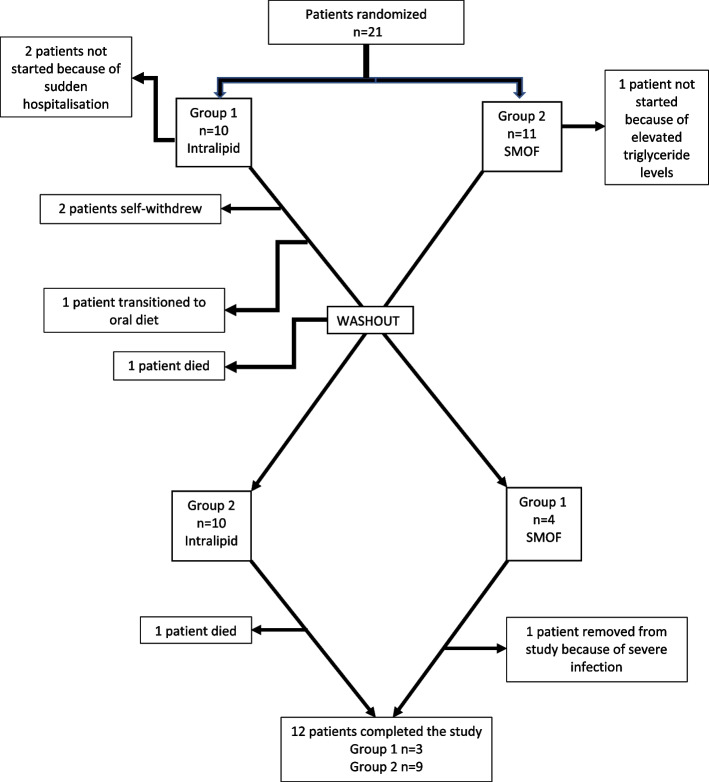
Study process flow chart

Sixty patients were assessed for eligibility, and a total of 21 patients were enrolled and randomized into two groups. The recruitment rate was 35%. The retention rate was 66.7% (14/21) at 7 months and 57% (12/21) at 13 months. Ten patients were randomized to group 1 (received SO first), and eleven were randomized to group 2 (received MO first). At baseline, these randomized groups were similar (Supplementary Table 1). Three patients who were randomized did not start the study, and 6 patients withdrew during the study. The reasons for withdrawal included self-withdrawal (*n* = 2), non-study-related death (*n* = 2), transition to oral diet (*n* = 1), and infection (*n* = 1). There was a significant difference in dropout between group 1 (*n* = 6) and group 2 (*n* = 3) (*P* = 0.03). Twelve subjects completed the study, 3 from group 1, and 9 from group 2 (see Fig. 2).

### Secondary patient-centered outcomes

#### Per protocol baseline characteristics

Baseline characteristics of the per-protocol population were similar between the two groups for all variables except for the type of vascular access, with more patients using peripherally inserted central catheters in group 2 and more using Hickman lines and port-o-caths in group 1 (Table 1).

**Table 1:** Baseline characteristics in the 6 months prior to enrolment of the per-protocol population of 12 patients who completed the study

	Group 1		Group 2		Group 1—Group 2
	*N* with data^c^	Mean (SD)	*N* with data^c^	Mean (SD)	Difference^a^ (95% CI)^b^
Total, *N*	3		9		
Age (years)	3	51.33 (21.96)	9	54.89 (7.10)	− 3.56 (− 55.69–48.58)
Female, *N* (%)	3	1 (33.3)	9	2 (22.2)	0.11 (− 0.42–0.72)
In 6 months prior to enrolment					
Surgery, *N* (%)	3	0 (0.0)	9	1 (11.1)	− 0.11 (− 0.48–0.56)
Unexpected hospitalization for infection, *N* (%)	3	0 (0.0)	9	3 (33.3)	− 0.33 (− 0.71–0.37)
New line infection, *N* (%)	3	0 (0.0)	9	3 (33.3)	− 0.33 (− 0.71–0.37)
New CRBSI, *N* (%)	3	0 (0.0)	9	1 (11.1)	− 0.11 (− 0.48–0.56)
New antibiotics prescribed for CRBSI, *N* (%)	3	0 (0.0)	9	1 (11.1)	− 0.11 (− 0.48–0.56)
Duration of antibiotic treatment (days)	3	0.00 (0.00)	9	2.11 (6.33)	− 2.11 (− 6.98–2.76)
Indication for HPN, *N* (%)					
Short bowel syndrome	3	2 (66.7)	9	5 (55.6)	0.11 (− 0.56–0.64)
GI dysmotility	3	0 (0.0)	9	0 (0.0)	N/A
GI obstruction	3	0 (0.0)	9	1 (11.1)	− 0.11 (− 0.48–0.56)
Chyle leak	3	0 (0.0)	9	1 (11.1)	− 0.11 (− 0.48–0.56)
Intolerance to enteral feeding	3	0 (0.0)	9	0 (0.0)	N/A
Other	3	1 (33.3)	9	2 (22.2)	0.11 (− 0.42–0.72)
Vascular access, *N* (%)					
Type of vascular access					
PICC	3	0 (0.0)	9	7 (77.8)	− 0.78 (− 0.97– − 0.03)
Hickman	3	2 (66.7)	9	2 (22.2)	0.44 (− 0.23–0.88)
Port-o-cath	3	1 (33.3)	9	0 (0.0)	0.33 (− 0.16–0.91)
Location of vascular access, *N* (%)					
Right	3	2 (66.7)	9	3 (33.3)	0.33 (− 0.35–0.81)
Left	3	1 (33.3)	9	6 (66.7)	− 0.33 (− 0.81–0.35)
Number of lumens, *N* (%)					
1	3	2 (66.7)	9	2 (22.2)	0.44 (− 0.23–0.88)
2	3	1 (33.3)	9	6 (66.7)	− 0.33 (− 0.81–0.35)
Nutritional status					
Weight (kg)	3	56.80 (8.93)	9	59.98 (15.75)	− 3.18 (− 20.82–14.47)
Body mass index (kg/m^2^)	3	21.90 (2.34)	9	22.90 (3.64)	− 1.00 (− 5.52–3.52)
Mid-arm circumference (cm)	3	27.07 (2.00)	7	26.99 (2.83)	0.08 (− 3.85–4.02)
Subjective global assessment, *N* (%)					
A	3	2 (66.7)	9	6 (66.7)	0.00 (− 0.65–0.53)
B	3	1 (33.3)	9	1 (11.1)	0.22 (− 0.30–0.80)
C	3	0 (0.0)	9	0 (0.0)	N/A
Parenteral nutrition					
Total energy (kcal/day)	3	1446.53 (364.38)	9	1244.30 (419.59)	202.23 (− 501.62–906.09)
Total energy (kcal/kg/day)	3	26.10 (8.74)	9	21.43 (7.38)	4.67 (− 13.13–22.47)
Amino acids (g/kg/day)	3	0.95 (0.10)	9	0.91 (0.26)	0.04 (− 0.20–0.28)
Lipids (g/kg/day)	3	0.75 (0.32)	9	0.60 (0.22)	0.15 (− 0.53–0.82)
Frequency of HPN (days/week)	3	5.33 (1.53)	9	5.33 (1.12)	0.00 (− 3.21–3.21)
Frequency of hydration (days/week)	3	2.33 (4.04)	9	0.89 (1.17)	1.44 (− 8.23–11.12)
Liver function test					
ALT (U/L)	3	40.00 (39.13)	9	23.33 (11.38)	16.67 (− 76.97–110.31)
AST (U/L)	3	23.00 (12.12)	9	29.11 (9.71)	− 6.11 (− 31.07–18.85)
ALP (U/L)	3	122.67 (14.64)	8	158.88 (104.50)	− 36.21 (− 124.25–51.84)
GGT (U/L)	3	29.33 (7.09)	9	49.11 (44.13)	− 19.78 (− 54.28–14.72)
Total bilirubin (umol/L)	3	9.67 (6.43)	9	8.78 (3.60)	0.89 (− 13.33–15.11)
Conjugated bilirubin (umol/L)	3	5.33 (4.04)	8	4.75 (1.98)	0.58 (− 8.47–9.64)
Albumin (g/L)	3	38.00 (3.61)	9	37.00 (3.67)	1.00 (− 6.08–8.08)
Lipid profile					
Total cholesterol (mmol/L)	3	2.47 (0.99)	9	3.03 (0.54)	− 0.56 (− 2.75–1.63)
Triglycerides (mmol/L)	3	0.93 (0.42)	9	1.06 (0.43)	− 0.13 (− 0.96–0.70)
Linoleic acid w-6	3	339.07 (159.55)	7	365.28 (90.39)	− 26.21 (− 370.48–318.06)
Alpha linolenic acid w-3	3	9.72 (6.39)	7	9.02 (2.67)	0.70 (− 13.82–15.22)
Eicosapentaenoic acid EPA w-3	3	20.77 (10.27)	7	18.60 (6.90)	2.17 (− 19.24–23.58)
Docosahexaenoic acid DHA w-3	3	80.24 (22.51)	7	85.81 (24.10)	− 5.57 (− 49.15–38.01)
Ratio w-6:w-3	3	37.57 (6.33)	6	40.43 (3.88)	− 2.87 (− 16.13–10.39)
Arachidonic acid	3	378.49 (134.16)	7	346.13 (87.03)	32.35 (− 249.36–314.06)
Coagulation markers					
APTT (s)	2	25.65 (0.07)	9	29.52 (3.95)	− 3.87 (− 6.91– − 0.83)
INR	3	1.10 (0.14)	8	1.36 (0.79)	− 0.26 (− 0.93–0.41)
Inflammation marker					
CRP (mg/L)	2	13.50 (12.02)	6	12.83 (23.60)	0.67 (− 35.27–36.62)
General biochemistry					
Hemoglobin (g/L)	3	124.00 (17.06)	9	111.89 (14.53)	12.11 (− 22.56–46.78)
White blood cells (× 109/L)	3	7.57 (2.99)	9	5.64 (2.32)	1.92 (− 4.27–8.12)
Platelets (× 109/L)	3	190.67 (72.02)	9	228.67 (93.73)	− 38.00 (− 175.93–99.93)
Sodium (mmol/L)	3	138.33 (1.15)	9	139.22 (2.77)	− 0.89 (− 3.47–1.70)
Potassium (mmol/L)	3	3.73 (0.55)	9	4.18 (0.48)	− 0.44 (− 1.56–0.67)
Bicarbonate (mmol/L)	3	25.33 (2.52)	9	23.22 (2.17)	2.11 (− 2.99–7.21)
Phosphate (mmol/L)	3	1.03 (0.19)	9	1.18 (0.21)	− 0.15 (− 0.52–0.22)
Calcium (mmol/L)	3	2.21 (0.09)	9	2.31 (0.07)	− 0.10 (− 0.28–0.09)
Magnesium (mmol/L)	3	0.76 (0.03)	9	0.83 (0.09)	− 0.08 (− 0.15–0.00)

*Abbreviations*: *CRBSI* Catheter-related bloodstream infection, *GI* Gastrointestinal, *PICC* Peripherally inserted central catheter, *HPN* Home parenteral nutrition, *ALT* Alanine aminotransferase, *AST* Aspartate aminotransferase, *ALP* Alkaline phosphatase, *GGT* Gamma-glutamyl transferase, *aPTT* (sec) Activated partial thromboplastin time, *INR* International normalized ratio, *CRP* C-reactive protein

^a^Difference in means for continuous variables and difference in proportions for categorical variables

^b^CIs were calculated using t-distribution for continuous variables and the exact method for categorical variables

^c^Number of participants in 6 months prior to enrollment in the per-protocol population

#### Potential treatment effect of the use of mo on biochemical parameters

After 6 months, the use of MO did not significantly impact any liver enzymes but resulted in a significant increase in the hemoglobin levels (*p* value 0.013) and WBC (*p* value 0.005) count compared to the use of SO. Additionally, the use of MO did not have an impact on coagulation markers, CRP, or the rest of the general biochemistry (Table 2).

**Table 2:** Potential treatment effect of the use of MO lipid on liver function tests, electrolytes, coagulation, and inflammation markers in the per-protocol population

Outcomes		**Least squares mean change from baseline**^**b**^		**Difference in mean change (SO–MO)**
	**n**^**a**^	**SO**	**MO**	**Least squares mean (95% CI)**
**Liver function test**				
ALT (U/L)	12	2.56	5.22	-2.67 (-15.89–10.56)
AST (U/L)	12	4.56	1.22	3.33 (-4.02–10.69)
ALP (U/L)	11	-0.33	-3.77	3.44 (-38.38–45.26)
GGT (U/L)	10	5.69	1.98	3.71 (− 3.54–10.97)
Total bilirubin (μmol/L)	12	1.06	0.44	0.61 (− 2.55–3.78)
Conjugated bilirubin (μmol/L)	9	− 0.33	− 0.50	0.17 (− 0.73 –1.06)
Albumin (g/L)	12	− 0.11	0.89	− 1.00 (− 2.83–0.83)
**Coagulation markers**				
APTT (s)	9	− 0.65	− 1.85	1.20 (− 2.62–5.02)
INR	9	− 0.10	− 0.15	0.05 (− 0.25–0.35)
**Inflammation marker**				
CRP (mg/L)	8	− 13.50	− 1.58	− 11.92 (− 49.06–25.22)
**General Biochemistry**				
Hemoglobin (g/L)	12	− 2.00	8.39	− 10.39 (− 18.08– − 2.69)
White blood cells (X10^9^/L)	12	− 1.77	1.16	− 2.93 (− 4.74– − 1.11)
Platelets (X10^9^/L)	12	− 1.89	10.11	− 12.00 (− 38.88–14.88)
Sodium (mmol/L)	12	− 0.61	− 1.50	.89 (− 2.05–3.83)
Potassium (mmol/L)	12	0.18	− 0.06	0.23 (− 0.07–0.54)
Bicarbonate (mmol/L)	12	0.89	− 0.17	1.06 (− 3.52–5.63)
Chlorine (mmol/L)	12	0.22	− 1.28	1.50 (− 1.64–4.64)
Phosphate (mmol/L)	12	0.06	0.07	− 0.00 (− 0.20–0.20)
Calcium (mmol/L)	12	− 0.02	0.02	− 0.04 (− 0.14–0.06)
Magnesium (mmol/L)	12	− 0.00	− 0.04	0.03 (− 0.03–0.10)

*Abbreviations*: *SO* Soybean oil, *MO* Mixed oil, *ALT* Alanine aminotransferase, *AST* Aspartate aminotransferase, *ALP* Alkaline phosphatase, *GGT* Gamma-glutamyl transferase, *aPTT (s)* Activated partial thromboplastin time, *INR* International normalized ratio, *CRP* C-reactive protein

*n*^*a*^ number of participants with all four measurements at baseline and after 6 months

^b^Baseline is defined as the time at study start for the first arm and month 7 for the second arm

Furthermore, using MO for 6 months did not affect the levels of total cholesterol and triglycerides when compared to SO; however, as expected, due to its composition, it led to a significant decrease in the levels of the alpha-linolenic acid ω-3 (*p* value 0.034) and a significant increase in the EPA (*p* value < 0.001) and DHA levels (*p* value 0.027) when compared to SO. There was also a trend towards decreased values of linoleic acid ω-6 with the use of MO, but the mean change for the SO and MO groups was not significantly different. The difference in the ω-6 to ω-3 ratio between the two treatment groups was not significant (Table 3). When adjusting for the order in which the lipids were administered to each group (the sequence), the time of administration of each lipid (the period), the type of lipid received (the treatment), and the change of the different liver enzymes from baseline (ALT, AST, ALP, and GGT), we could detect the impact of group sequence on the ω-6 to ω-3 ratio vs. ALP and ω-6 to ω-3 ratio vs. conjugated bilirubin (Supplementary Material Table 2).

**Table 3:** Potential treatment effect of MO lipid to SO lipid use on the lipid profile and fatty acid levels in the per-protocol population

**Outcomes**		**Least squares mean change from baseline**^**b**^		**Difference in mean change (SO-MO)**
	n^a^	**SO**	**MO**	**Least squares mean (95% CI)**
**Lipid profile**				
Total cholesterol (mmol/L)	9	0.10	0.10	0.00 (− 0.65–0.66)
Triglycerides (mmol/L)	11	0.20	0.36	− 0.16 (− 0.56–0.24)
**Fatty acid profile**				
Linoleic acid ω-6	9	49.46	− 123.00	172.16 (− 5.62–349.94)
Alpha linolenic acid (ω-3)	9	1.81	− 2.91	4.72 (0.47–8.97)
Eicosapentaenoic acid (EPA ω-3)	9	− 9.89	50.16	− 60.05 (− 74.72– − 45.39)
Docosahexaenoic acid (DHA ω-3)	9	− 12.1	73.80	− 85.92 (− 158.99– − 12.85)
Ratio ω-6–ω-3	8	− 2.38	− 0.79	− 1.59 (− 11.37–8.20)
Arachidonich acid	9	39.38	− 98.60	138.01 (− 64.47–340.49)

*Abbreviations*: *SO* Soybean oil, *MO* Mixed oil

^a^Number of participants with all four measurements at baseline and months 6, 7, and 13

^b^Baseline is defined as the time at study start for the first arm and month 7 for the second arm

Next, we investigated the correlation between the change in ω-6 to ω-3 ratio and the changes in the levels of ALT, AST, ALP, GGT, CRP, hemoglobin, and WBC counts between MO and SO (data not shown). Our analysis demonstrated a statistically significant strong negative correlation between the changes in the ω-6 to ω-3 ratio and the changes in the hemoglobin values (p = 0.049, rho =  − 0.66) when using SO but not when using MO. No other correlation was demonstrated for the ω-6 to ω-3 ratio.

#### Interaction between treatment (SO/MO)

We compared the treatment effect of using MO and SO on correlations of changes in lipid profile values (ω-6 to ω-3 ratio, ω-6 and ω-3 levels) and changes in ALT, AST, ALP, GGT, CRP, hemoglobin levels, and WBC count. Our analysis demonstrated heterogeneity of the treatment effect when looking at the correlation of changes in ω-3 levels and changes in GGT values with a larger negative correlation in SO than MO (*P* = 0.028). There was no statistically significant heterogeneous treatment effect for all the other correlations evaluated between MO and SO (see Supplementary Material Figs. 1–5).

#### Clinical outcomes and complications

In this pilot study overall, clinical outcomes and complications were comparable between SO and MO, except for a trend towards a longer duration of antibiotic therapy with SO use. A similar number of patients had adverse events, unexpected hospitalizations, use of antibiotics, new infections, and changes in vascular access between the two lipids. No new CRBSI was reported for either lipid (Table 4).

**Table 4:** Clinical outcomes and complications in the 6-month period on each lipid solution in per-protocol population

	SO		MO		SO-MO
	*N* with data^b^	*N* (%)	*N* with data^b^	*N* (%)	Difference^a^ (95% CI)
Total, *N*	12		12		
New CRBSI	12	0 (0.0)	12	0 (0.0)	N/A
New infections including CRSBI	12	4 (33.3)	12	3 (25.0)	0.08 (− 0.20–0.36)
Change of vascular access	12	3 (25.0)	12	1 (8.3)	0.17 (− 0.15–0.48)
Use of antibiotics	12	6 (50.0)	12	2 (16.7)	0.33 (− 0.02–0.69)
Duration antibiotics therapy (days) LS Mean	12	4.39 (6.99)	11	0.17 (2.11)	4.22 (− 0.84–9.28)
Uexpected hospitalization	12	2 (16.7)	12	2 (16.7)	0.00 (− 0.33–0.33)
Surgery	12	1 (8.3)	12	0 (0.0)	N/A
Death	12	0 (0.0)	12	0 (0.0)	N/A
Adverse event	12	6 (50.0)	12	4 (33.3)	0.17 (− 0.29–0.62)
Serious adverse event	12	0 (0.0)	12	1 (8.3)	N/A
Serious adverse event probably related to the intervention	12	0 (0.0)	12	0 (0.0)	N/A

N/A since no statistics were computed if no clinical outcomes or complications occurred for both treatments

*Abbreviations*: *SO* Soybean oil, *MO* Mixed oil, *CRBSI* Catheter-related bloodstream infection

^a^Risk difference for all the binary outcomes and mean difference for duration antibiotics therapy

^b^Number of participants who completed the study with at least one episode of events was counted

### Sensitivity analysis

We also performed a sensitivity analysis with the intention to treat population (Supplementary Table 3) and the results confirmed our findings of the primary efficacy, i.e., the results are the same using per-protocol analysis and intention to treat analysis in terms of significance.

## Discussion

Overall, this pilot trial demonstrated that the use of a prospective double-blind, crossover, and randomized trial design was not feasible to conduct in the HPN population due to difficulties in recruiting and retaining patients. Additionally, this study found that in HPN patients, MO use as lipid emulsion versus SO did not significantly impact liver enzymes or overall biochemical parameters or clinical outcomes despite changes in plasma fatty acid composition that reflected the different types of lipid emulsions. The only significant change detected was an increase in hemoglobin and white blood cells with MO. One possible explanation is that changes in the blood cell membrane fatty acid composition may affect blood cell adhesion, aggregation, red blood cell deformation, and cell membrane elasticity [29–31], contributing to the improvement in WBC and hemoglobin levels in the MO group versus the SO group.

This study presented several challenges in this complex HPN population that will need to be taken into account for a future randomized trial.

### Recruitment and dropouts

Recruitment and retention for this study were challenging. This is a small, specialized patient population. Our program is one of the largest in Canada with a total of 65 HPN patients dispersed in the province of Ontario where long travel distance for clinic or study visits is an issue considering that at least half of our HPN patients live more than 2 h of driving distance from our hospital and are followed by telehealth. Therefore, these patients could not be approached for the study as they could not be assessed face-to-face and provided blood for specialized laboratory tests like plasma-free fatty acid composition. This is a situation that is similar to other HPN programs across Canada. In addition, many patients have complex medical issues that make them less enthusiastic to participate or have reduced mobility that make it challenging to come for frequent study visits. Therefore, this was a factor that affected recruitment in this crossover study considering the necessity of having several visits (3 per arm; total 6 visits) for bloodwork. In order to get a larger number of patients, a future trial will need to include several centers, and considering similar patient population and distances, a parallel design may be less complicated versus a crossover, as it would reduce study duration and the number of study visits.

There were also several dropouts, some due to complications and deaths, with higher dropouts in the first period of Group 1 which corresponded to the first arm using SO. Considering that all patients were already on SO before starting the study, and that this arm was just a continuation of the same lipid emulsion, the dropout may not likely be related to SO versus MO. High dropouts could be due to the complex care and high risk of complications associated with HPN in this patient population but there were no major differences in the baseline characteristics of enrolled subjects prior to starting the trial that could have contributed to higher dropouts in those starting with SO (Group 1) (Supplementary Table 1).

The high dropout rate resulted in a significant reduction in the number of subjects in Group 1 and it reduced the power of our study. For a large multicenter randomized trial, factors that may affect dropouts and complications, such as demographics, HPN indications, co-morbidities, and type of central venous access, would be balanced between groups. Access to care is also important, especially if long-distance needs to be traveled. In this case, monetary compensation for traveling expenses could decrease dropout rates.

The HPN population, the number of hospitalizations, line sepsis, and deaths reported in this study was comparable to other published literature [12, 14, 23]. HPN patients are generally complex, often with significant underlying diseases that can require hospitalization and a high risk of central line infection that can lead to complications such as endocarditis, septic shock, and death [32]. Indeed, during the study, two participants died and one developed endocarditis that required prolonged antibiotic treatment.

### Sample size calculation

Therefore, the sample size calculation will need to take into account the high drop-out rate. From the pilot study, the overall high drop-out rate was 9/21 (43%). Considering our results and using the same cross-over design with similar laboratory parameters, we calculated sample sizes with selected variables reflecting liver function and inflammation, to inform potential future studies in detecting the presence of treatment effects (see Table 5).

**Table 5:** Sample size required for potential future cross-over studies: number of pairs required based on the data observed

Power	80%	85%	90%
ALT	72	83	97
ALP	402	460	536
CRP	27	30	36
WBC	3	3	4

The parameters used in this calculation are all from the study results (Table 2), the calculations are based on a two-sided test at a 5% significance level and considering a 43% drop-out rate

*ALP* alkaline phosphatase, *ALT* alanine transaminase, *CRP* C-reactive protein, *WBC* white blood cells

Given the difficulties related to high dropout rates and numerous visits for the cross-over study design [33], we also calculated the sample size needed for a parallel design using the same variables and the estimates from the present study (Table 6). The sample size calculation was based on the first phase of the study and is much larger than with the crossover design. In a parallel design trial, all patients would receive only one of the allocated treatments, require fewer visits, possibly reducing dropout rate, and the two groups would be compared to each other. The study could assess longer treatment duration. On the other hand, in a crossover trial, all patients would receive all the allocated treatments and act as their own controls but it would require more visits with a higher risk of dropouts and, for the same treatment duration, the study would be longer than a parallel design. Another factor when comparing the two designs is the marked heterogeneity of HPN patients: there would be a larger influence of confounding covariates in a parallel design trial versus a crossover trial design where the patient is his/her own control, providing smaller variability than the in the between-patient analysis required for a parallel design. Therefore, a parallel design trial requires a much larger number of patients compared to a crossover study to achieve the same power. In a recent published protocol for an ongoing 8-week randomized controlled trial comparing MO to another type of lipid emulsion in a similar HPN population, a parallel design was chosen to assess combined changes in liver parameters (ALT, AST, bilirubin). The sample size was calculated to be 160 patients (80 in each group) [5, 34] using a power of 0.8, a significance set at 0.025 and a drop-out rate of 20%. However, the authors did not provide the reference for the study used to calculate the sample size and did not clarify if this was based on in-patients or HPN patients’ results. Based on our estimates (Table 6), it is possible that this study does not have sufficient power.

**Table 6:** Sample size required for a parallel study design based on the data observed

Power	80%	85%	90%
ALT	1050	1200	1404
ALP	908	1038	1214
CRP	104	118	138
WBC	16	18	20

The calculations are based on a two-sided test at a 5% significance level and the sample size is for the total number of patients required

*ALP* alkaline phosphatase, *ALT* alanine transaminase, *CRP* C-reactive protein, *WBC* white blood cells

### Study duration and patient selection

Another consideration is the study duration and HPN regimen. A 6-month duration to assess the effect of MO on liver enzymes should be sufficient based on the literature on HPN [11, 23]. If only evaluating safety and tolerance, a previous study used a 4-week period to find that SO, medium-chain triglycerides, and olive and fish oil emulsion were all well tolerated and safe in 73 IF patients on long-term HPN [11]. Another study used 60 days for 32 adults on long-term HPN where SO was changed to either MO or olive oil [23]. Results found that both were well tolerated and that MO did not alter liver function markers although olive-oil-based lipid emulsion decreased some liver function tests. However, the response could depend on baseline liver enzyme levels: individuals with higher liver enzymes may have a higher magnitude of effect than those with lower levels. In previous studies [14, 35], patients with at least 1.5 to 2 times normal liver enzymes at baseline were recruited if liver enzymes were the main outcome. On the other hand, for clinical outcomes, a 6-month duration will not be sufficient to assess the rate of hospitalization, antibiotic use, or mortality. Our previous multicenter cohort study was of a 2-year duration with 120 subjects, which made it unique compared to other smaller HPN studies in the literature [26]. Clinical outcomes of interest for HPN populations were poorly studied previously, including the number of hospitalizations, the number of hospitalizations related to HPN, and the incidence of line sepsis per 1000 catheter days in each group using SO and MO. The results showed an increased risk of hospitalization in HPN patients receiving MO lipid emulsion [26]. Therefore, if hospitalization is to be used as the main outcome, a 2-year duration with a least the same number of subjects using a parallel design is more appropriate for a larger randomized controlled trial and this should also be multicenter.

In addition, HPN patients have different PN regimens as opposed to inpatients who generally receive PN daily because of poor oral intake. In our HPN population, patients can receive 3 to 7 days of PN weekly, with either a hyperphagic diet if short bowel syndrome, with variable absorption being present, or no oral intake if severe dysmotility or; intermittent oral intake if other indications like surgical complications. These various HPN regimens and oral intake will likely impact on the change in fatty acid composition that is required to have a certain biological effect. In our pilot study, we detected significant differences in fatty acid composition, reflecting the different types of lipids administered, despite the small number of patients, different regimens, and some hospitalizations that may require delaying or abstaining from PN due to line infection. However, the magnitude of the effect from different lipid emulsions could be greater if only patients on the 7-day regimen with poor intake are used for a larger randomized trial. The lack of effect on liver enzymes could be due to insufficient infusion of MO in our patients with various HPN regimens, despite significant changes in plasma fatty acid composition. Another possibility is that the liver enzymes were not sufficiently abnormal at baseline to detect a difference over the 6-month period. With a larger number of patients, as suggested by our sample size calculations, the heterogeneity of our HPN population may not be as significant if the two parallel groups are well balanced in terms of causes of chronic IF and HPN regimen.

Finally, another challenge of conducting a large multicenter trial will be to switch many HPN patients back to SO from MO lipid emulsions considering that many HPN programs, influenced by the studies reporting positive findings in the pediatric population [36–38], in hospitalized adult patients, [12] or small number of HPN patients, switched their entire patient population to MO lipids [14, 23, 25, 26].

Therefore, conducting a large trial would require some degree of acceptance by HPN programs that more data are required to prescribe one lipid emulsion over another, especially in view of the results of our prospective cohort study [26].

### Feasibility of a definitive trial

A multicenter, randomized, double-blinded, parallel trial of at least a 2-year duration comparing MO to SO should be feasible considering the number of HPN patients in Canada. We have an established network of HPN programs already participating in the Canadian HPN Registry (28). Based on the recent number of potentially eligible patients from our previous cohort study [26], we estimate that about 850 out of 980 patients enrolled in our registry could be approached, as this excludes those with metastatic disease and poor prognosis. The survival rate in our patient population without active malignancy is about 80% at 5 years [39–41]. Therefore, a 5-year duration is also feasible if we want to better capture clinical outcomes such as hospitalization rate and mortality. Patients with low-risk survival, such as those with active malignancies, would need to be excluded. If liver enzymes are chosen as the main outcomes, patients with elevated liver enzymes of at least 1.5 to 2 times normal should then be selected; this would restrict the number of potential subjects. In addition, other potential difficulties include the elevation of liver enzymes due to sepsis, cholecystitis, or common bile duct stones, which are not infrequent in this population and are independent of the type of lipid emulsion. Therefore, we favor a primary clinical outcome such as hospitalization rate per year and also include PN- and non-PN-related hospitalization, line sepsis per 1000/catheter days, antibiotic use, antibiotic days, and mortality, similar to our prospective cohort study [26]. In addition, considering the risk of non-alcoholic fatty liver disease (NAFLD) and intestinal failure-associated liver disease (IFALD) in the HPN population, assessments of liver fibrosis (eg FIB-4, APRI, Fibroscan) could be of benefit as it is associated with detrimental outcomes [42]. Furthermore, study visits should correspond to clinic visits every 6 months to improve recruitment and reduce dropouts. It should include patients who are followed by telehealth. When considering inclusion and exclusion criteria, those on GLP-2 agonists would need to be excluded because of the potential confounding effect of HPN and lipid emulsion reduction over time, while on GLP2. The Canadian HPN Registry has presently 34 patients on GLP-2 agonists who are gradually reducing or being weaned from HPN (manuscript submitted): these would not be able to participate in a future trial on lipid emulsions. Finally, considering that many patients are on a variety of oils for their lipid emulsions (SO, MO, or olive oil), a run-in period with SO of 3-month duration should be considered so that every participant begins the study with a similar baseline regarding plasma or red blood cell fatty acid composition that would reflect the same type of lipid emulsion before randomization. If telehealth patients cannot provide blood for this measurement, the fatty acid composition can be performed in a subgroup of patients.

## Conclusion

In conclusion, our 6-month pilot study using a double-blind crossover design was not feasible to conduct in the HPN population due to difficulties in recruiting and retaining patients. In addition, this study did not show any significant effect of MO versus SO on liver enzymes or most of the biochemical and clinical parameters. There were several challenges in conducting such a trial which was inherent to the complexity and the heterogeneity of the patient population and their HPN regimen. This will need to be taken into account if a large multicenter, double-blind, randomized controlled trial is planned. A parallel design with a study duration of at least 2 years should be considered to better capture clinical outcomes.

## Supplementary Information

**Additional file 1:** **Table S1.** Baseline characteristics (Intention-to-treat population: all patients randomized in the study). **Table S2.** Effects of sequence, period, treatment, and liver enzymes on the ω-6:ω-3 ratio (PP population): ANCOVA model for repeated measures with sequence, period, treatment and liver enzyme as fixed effects, and patient as a random effect. **Table S3.** Sensitivity Analysis with Intention to Treat Population.

**Additional file 2: Supplementary Material Figure 1. **Interaction betweenTreatment (Soybean Oil/Mixed Oil) and the change in liver enzymes on the changein ratio w-6: w-3. **Supplementary Material Figure 2. **Interaction between Treatment (Soybean Oil/MixedOil) and the change in liver enzymes on the change inOmega 6. **Supplementary Material Figure 3.** Interaction between Treatment (Soybean Oil/MixedOil) and the change inliver enzymes on the change in Omega 3. **Supplementary Material Figure 4. **Interaction between Treatment (Soybean Oil/MixedOil) and the change in liver enzymes on the change inEPA. **Supplementary Material Figure 5. **Interaction between Treatment (Soybean Oil/MixedOil) and the change in liver enzymes on the change inDHA.

## Data Availability

The datasets used and/or analyzed during the current study are available from the corresponding author on reasonable request.
