# Supplementary material for: Comparing mixed oil to soybean oil lipid emulsion in patients on home parenteral nutrition: a pilot prospective double-blind, crossover, randomized trial
Source: Pilot Feasibility Stud. 2023 Apr 20;9:64. doi: 10.1186/s40814-023-01295-1 (PMC10116729; doi:10.1186/s40814-023-01295-1)
Supplement: Supplementary file 1 — Additional file 1: Table S1. Baseline characteristics (Intention-to-treat population: all patients randomized in the study). Table S2. Effects of sequence, period, treatment, and liver enzymes on the ω-6:ω-3 ratio (PP population): ANCOVA model for repeated measures with sequence, period, treatment and liver enzyme as fixed effects, and patient as a random effect. Table S3. Sensitivity Analysis with Intention to Treat Population. [file 40814_2023_1295_MOESM1_ESM.docx]

**Supplementary Material:**

**Table 1:** Baseline characteristics (Intention-to-treat population: all patients randomized in the study)

|  | | Group 1 | | Group 2 | | | Group 1 - Group 2 | |
| --- | --- | --- | --- | --- | --- | --- | --- | --- |
|  |  | N with data | Mean (SD) | N with data | Mean (SD) | | Difference* (95% CI)** | |
| Total N | | 7 |  | 10 |  | |  | |
| Age (years) | | 7 | 53.00 ( 14.69 ) | 10 | 54.10 ( 7.14 ) | | -1.10 ( -14.92 - 12.72 ) | |
| Female N(%) | | 7 | 2 ( 28.6 ) | 10 | 2 ( 20.0 ) | | 0.09 ( -0.37 - 0.54 ) | |
| In the 6 months prior to enrolment | |  |  |  |  | |  | |
| Surgery N(%) | | 7 | 0 ( 0.0 ) | 10 | 1 ( 10.0 ) | | -0.10 ( -0.45 - 0.31 ) | |
| Unexpected Hospitalization for infection N(%) | | 7 | 2 ( 28.6 ) | 10 | 3 ( 30.0 ) | | -0.01 ( -0.46 - 0.47 ) | |
| New line infection N(%) | | 7 | 2 ( 28.6 ) | 10 | 3 ( 30.0 ) | | -0.01 ( -0.46 - 0.47 ) | |
| New CRBSI N(%) | | 7 | 1 ( 14.3 ) | 10 | 1 ( 10.0 ) | | 0.04 ( -0.33 - 0.48 ) | |
| New antibiotics prescribed for CRBSI N(%) | | 7 | 1 ( 14.3 ) | 10 | 1 ( 10.0 ) | | 0.04 ( -0.33 - 0.48 ) | |
| Duration of antibiotic treatment (days) | | 7 | 0.71 ( 1.89 ) | 10 | 1.90 ( 6.01 ) | | -1.19 ( -5.64 - 3.26 ) | |
| Indication for HPN N(%) | |  |  |  |  | |  | |
| Short Bowel Syndrome | | 7 | 3 ( 42.9 ) | 10 | 5 ( 50.0 ) | | -0.07 ( -0.53 - 0.42 ) | |
| GI dysmotility | | 7 | 0 ( 0.0 ) | 10 | 0 ( 0.0 ) | | N/A | |
| GI obstruction | | 7 | 2 ( 28.6 ) | 10 | 1 ( 10.0 ) | | 0.19 ( -0.23 - 0.61 ) | |
| Chyle leak | | 7 | 0 ( 0.0 ) | 10 | 1 ( 10.0 ) | | -0.10 ( -0.45 - 0.31 ) | |
| Intolerance to enteral feeding | | 7 | 1 ( 14.3 ) | 10 | 0 ( 0.0 ) | | 0.14 ( -0.18 - 0.58 ) | |
| Other | | 7 | 2 ( 28.6 ) | 10 | 3 ( 30.0 ) | | -0.01 ( -0.46 - 0.47 ) | |
| Vascular Access N(%) | |  |  |  |  | |  | |
| Type of vascular access | |  |  |  |  | |  | |
| PICC | | 7 | 2 ( 28.6 ) | 10 | 7 ( 70.0 ) | | -0.41 ( -0.78 - 0.10 ) | |
| Hickman | | 7 | 4 ( 57.1 ) | 10 | 3 ( 30.0 ) | | 0.27 ( -0.23 - 0.69 ) | |
| Port-o-cath | | 7 | 1 ( 14.3 ) | 10 | 0 ( 0.0 ) | | 0.14 ( -0.18 - 0.58 ) | |
| Location of vascular access N(%) | |  |  |  |  | |  | |
| Right | | 7 | 5 ( 71.4 ) | 10 | 4 ( 40.0 ) | | 0.31 ( -0.19 - 0.71 ) | |
| Left | | 7 | 2 ( 28.6 ) | 10 | 6 ( 60.0 ) | | -0.31 ( -0.71 - 0.19 ) | |
| Number of lumens N(%) | |  |  |  |  | |  | |
| 1 | | 7 | 3 ( 42.9 ) | 10 | 3 ( 30.0 ) | | 0.13 ( -0.35 - 0.58 ) | |
| 2 | | 7 | 4 ( 57.1 ) | 10 | 6 ( 60.0 ) | | -0.03 ( -0.50 - 0.44 ) | |
| Nutritional Status | |  |  |  |  | |  | |
| Weight (kg) | | 7 | 59.17 ( 11.90 ) | 10 | 58.45 ( 15.62 ) | | 0.72 ( -13.53 - 14.97 ) | |
| Body mass index (kg/m2) | | 7 | 22.03 ( 2.98 ) | 10 | 22.28 ( 3.95 ) | | -0.25 ( -3.84 - 3.34 ) | |
| Mid arm circumference (cm) | | 6 | 27.50 ( 2.67 ) | 8 | 26.24 ( 3.37 ) | | 1.26 ( -2.26 - 4.78 ) | |
| Subjective Global Assessment N(%) | |  |  |  |  | |  | |
| A | | 7 | 5 ( 71.4 ) | 10 | 6 ( 60.0 ) | | 0.11 ( -0.37 - 0.55 ) | |
| B | | 7 | 1 ( 14.3 ) | 10 | 2 ( 20.0 ) | | -0.06 ( -0.47 - 0.41 ) | |
| C | | 7 | 0 ( 0.0 ) | 10 | 0 ( 0.0 ) | | N/A | |
| Parenteral Nutrition | |  |  |  |  | |  | |
| Total Energy (kcal/day) | | 7 | 1496.57 ( 474.90 ) | 10 | 1316.66 ( 457.01 ) | | 179.91 ( -318.99 - 678.81 ) | |
| Total Energy (kcal/kg/day) | | 7 | 25.54 ( 8.19 ) | 10 | 23.69 ( 9.97 ) | | 1.85 ( -7.59 - 11.30 ) | |
| Amino Acids (g/kg/day) | | 7 | 1.07 ( 0.28 ) | 10 | 0.99 ( 0.35 ) | | 0.09 ( -0.24 - 0.41 ) | |
| Lipids (g/kg/day) | | 7 | 0.70 ( 0.24 ) | 10 | 0.63 ( 0.22 ) | | 0.07 ( -0.18 - 0.32 ) | |
| Frequency of HPN (days/week) | | 7 | 5.71 ( 1.70 ) | 10 | 5.50 ( 1.18 ) | | 0.21 ( -1.45 - 1.87 ) | |
| Frequency of Hydration (days/week) | | 7 | 2.57 ( 3.36 ) | 10 | 0.80 ( 1.14 ) | | 1.77 ( -1.35 - 4.89 ) | |
| Liver Function Test | |  |  |  |  | |  | |
| ALT (U/L) | | 7 | 31.14 ( 24.22 ) | 10 | 22.00 ( 11.53 ) | | 9.14 ( -13.62 - 31.90 ) | |
| AST (U/L) | | 7 | 25.00 ( 7.57 ) | 10 | 28.50 ( 9.36 ) | | -3.50 ( -12.30 - 5.30 ) | |
| ALP (U/L) | | 7 | 130.00 ( 32.02 ) | 9 | 150.11 ( 101.23 ) | | -20.11 ( -100.02 - 59.79 ) | |
| GGT (U/L) | | 7 | 39.57 ( 36.28 ) | 10 | 47.00 ( 42.14 ) | | -7.43 ( -48.38 - 33.52 ) | |
| Total Bilirubin (umol/L) | | 7 | 8.38 ( 4.84 ) | 10 | 9.00 ( 3.46 ) | | -0.62 ( -5.35 - 4.12 ) | |
| Conjugated Bilirubin (umol/L) | | 7 | 3.86 ( 3.13 ) | 9 | 4.89 ( 1.90 ) | | -1.03 ( -4.05 - 1.99 ) | |
| Albumin (g/L) | | 7 | 38.00 ( 2.38 ) | 10 | 37.40 ( 3.69 ) | | 0.60 ( -2.54 - 3.74 ) | |
| Lipid profile | |  |  |  |  | |  | |
| Total cholesterol (mmol/L) | | 7 | 3.18 ( 1.55 ) | 10 | 3.05 ( 0.51 ) | | 0.13 ( -1.31 - 1.57 ) | |
| Triglycerides (mmol/L) | | 7 | 0.98 ( 0.57 ) | 10 | 1.04 ( 0.41 ) | | -0.06 ( -0.61 - 0.50 ) | |
| Linoleic Acid w-6 | | 4 | 308.75 ( 143.69 ) | 8 | 375.62 ( 88.65 ) | | -66.88 ( -280.76 - 147.00 ) | |
| Alpha Linolenic Acid w-3 | | 4 | 8.81 ( 5.52 ) | 8 | 9.39 ( 2.68 ) | | -0.58 ( -8.92 - 7.77 ) | |
| Eicosapentaenoic acid EPA w-3 | | 4 | 18.37 ( 9.67 ) | 8 | 18.71 ( 6.40 ) | | -0.34 ( -14.68 - 13.99 ) | |
| Docosahexaenoic acid DHA w-3 | | 4 | 88.28 ( 24.42 ) | 8 | 87.10 ( 22.61 ) | | 1.18 ( -35.03 - 37.38 ) | |
| Ratio w-6:w-3 | | 4 | 37.13 ( 5.24 ) | 7 | 40.02 ( 3.71 ) | | -2.89 ( -10.65 - 4.87 ) | |
| Arachidonic Acid | | 4 | 340.57 ( 133.23 ) | 8 | 360.21 ( 89.87 ) | | -19.63 ( -217.05 - 177.79 ) | |
| Coagulation markers | |  |  |  |  | |  | |
| APTT (sec) | | 6 | 27.53 ( 3.10 ) | 10 | 29.57 ( 3.73 ) | | -2.04 ( -5.80 - 1.73 ) | |
| INR | | 7 | 1.05 ( 0.10 ) | 9 | 1.34 ( 0.74 ) | | -0.29 ( -0.87 - 0.28 ) | |
| Inflammation marker | |  |  |  |  | |  | |
| CRP (mg/L) | | 4 | 10.25 ( 8.54 ) | 6 | 12.83 ( 23.60 ) | | -2.58 ( -27.71 - 22.55 ) | |
| General Biochemistry | |  |  |  |  | |  | |
| Hemoglobin (g/L) | | 7 | 119.00 ( 14.93 ) | 10 | 110.20 ( 14.70 ) | | 8.80 ( -7.01 - 24.61 ) | |
| White blood cells (x109/L) | | 7 | 6.83 ( 2.60 ) | 10 | 5.39 ( 2.33 ) | | 1.44 ( -1.24 - 4.12 ) | |
| Platelets (x109/L) | | 7 | 237.71 ( 101.72 ) | 10 | 223.70 ( 89.75 ) | | 14.01 ( -90.15 - 118.18 ) | |
| Sodium (mmol/L) | | 7 | 137.86 ( 1.46 ) | 10 | 139.10 ( 2.64 ) | | -1.24 ( -3.39 - 0.90 ) | |
| Potassium (mmol/L) | | 7 | 4.07 ( 0.55 ) | 10 | 4.14 ( 0.47 ) | | -0.07 ( -0.63 - 0.49 ) | |
| Bicarbonate (mmol/L) | | 7 | 26.14 ( 2.19 ) | 10 | 23.30 ( 2.06 ) | | 2.84 ( 0.56 - 5.13 ) | |
| Phosphate (mmol/L) | | 6 | 1.10 ( 0.18 ) | 10 | 1.24 ( 0.27 ) | | -0.14 ( -0.38 - 0.10 ) | |
| Calcium (mmol/L) | | 7 | 2.21 ( 0.06 ) | 10 | 2.31 ( 0.06 ) | | -0.09 ( -0.16 - -0.03 ) | |
| Magnesium (mmol/L) | | 7 | 0.79 ( 0.07 ) | 10 | 0.83 ( 0.09 ) | | -0.04 ( -0.12 - 0.04 ) | |
|  |  |  |  |  |  |  |  |  |
| * Mean difference for continuous variables and proportion difference for categorical variables. | | | | | |  |  |  |
| ** CIs were calculated using t-distribution for continuous variables and exact method for categorical variables. | | | | | | | |  |

CRBSI, Catheter-Related Bloodstream Infection; GI, gastrointestinal; HPN, Home Parenteral Nutrition.

**Table 2.** Effects of sequence, period, treatment, and liver enzymes on the ω-6:ω-3 ratio (PP population): ANCOVA model for repeated measures with sequence, period, treatment and liver enzyme as fixed effects, and patient as a random effect

| Effects | N with data | Estimate (95% CI) | | | | | |
| --- | --- | --- | --- | --- | --- | --- | --- |
|  |  | Sequence | Period | Treatment | | | Lipid enzyme** |
| ANOVA Models for repeated measures* |  |  |  |  | | |  |
| Model 1: O6O3 ratio vs. ALT | 8 | -9.81 ( -20.91 - 1.30 ) | 3.47 ( -7.49 - 14.43 ) | -1.82 ( -12.89 - 9.25 ) | | | -0.06 ( -0.46 - 0.35 ) |
| Model 2: O6O3 ratio vs. AST | 8 | -11.40 ( -24.85 - 2.05 ) | 4.17 ( -6.28 - 14.61 ) | -2.35 ( -13.07 - 8.36 ) | | | 0.17 ( -0.67 - 1.02 ) |
| Model 3: O6O3 ratio vs. ALP | 8 | -11.33 ( -22.48 - -0.17 ) | 3.51 ( -6.87 - 13.90 ) | -1.62 ( -12.00 - 8.77 ) | | | -0.04 ( -0.16 - 0.07 ) |
| Model 4: O6O3 ratio vs. GGT | 6 | -10.33 ( -29.18 - 8.53 ) | 5.69 ( -19.24 - 30.63 ) | -2.56 ( -21.38 - 16.25 ) | | | 0.13 ( -1.13 - 1.39 ) |
| Model 5: O6O3 ratio vs. Conjugated Bilirubin | 7 | -14.29 ( -26.13 - -2.45 ) | 5.11 ( -7.05 - 17.27 ) | -1.69 ( -13.67 - 10.30 ) | | | 4.33 ( -2.80 - 11.46 ) |
| * Each model included O6:O3 ratio as the reponse variable vs. sequence, period, treatment, and each lipid as the explanatory variables. | | | | | |  |  |
| ** ALT for Model 1; AST for Model 2; ALP for Model 3; GGT for Model 4; Conjugated Bilirubin for Model 5 | | | | |  |  |  |

ω-6, linoleic acid; ω-3, alpha linoleic acid; PP, per-protocol; ANCOVA, analysis of covariance; ALT, Alanine Aminotransferase; AST, Aspartate Aminotransferase; ALP, Alkaline Phosphatase; GGT, Gamma-Glutamyl Transferase.

**Table 3:** Sensitivity Analysis with Intention to Treat Population

|  | Least Squares Mean Change from Baseline** | | Difference in Mean Change (Intralipid-SMOF) |
| --- | --- | --- | --- |
| Outcomes | Intralipid | SMOF | Least Squares Mean (95% CI) |
| Liver function test |  |  |  |
| ALT (U/L) | -1.11 | 5.37 | -6.49( -17.78- 4.81) |
| AST (U/L) | 1.34 | 1.32 | 0.01( -7.33- 7.36) |
| ALP (U/L) | -3.20 | -4.01 | 0.81( -35.83- 37.45) |
| GGT (U/L) | 18.07 | 14.47 | 3.61( -3.66- 10.88) |
| Total Bilirubin (umol/L) | 0.31 | 0.18 | 0.13( -2.74- 3.00) |
| Conjugated Bilirubin (umol/L) | -0.09 | -0.27 | 0.18( -0.74- 1.11) |
| Albumin (g/L) | -0.59 | 0.56 | -1.15( -3.01- 0.71) |
| Coagulation markers |  |  |  |
| APTT (sec) | -0.03 | -2.22 | 2.19( -1.07- 5.45) |
| INR | -0.06 | -0.10 | 0.05( -0.21- 0.30) |
| Inflammation marker |  |  |  |
| CRP (mg/L) | -9.73 | -1.69 | -8.04( -49.55- 33.47) |
| General Biochemistry |  |  |  |
| Hemoglobin (g/L) | -3.11 | 7.63 | -10.75( -18.44- -3.06) |
| White blood cells (x109/L) | -0.99 | 1.35 | -2.33( -4.11- -0.55) |
| Platelets (x109/L) | 7.71 | 12.37 | -4.66( -29.74- 20.42) |
| Sodium (mmol/L) | -0.61 | -1.50 | 0.89( -1.59- 3.37) |
| Potassium (mmol/L) | 0.09 | -0.04 | 0.12( -0.15- 0.39) |
| Bicarbonate (mmol/L) | -0.13 | 0.45 | -0.58( -4.66- 3.51) |
| Chlorine (mmol/L) | 0.07 | -1.41 | 1.49( -1.31- 4.28) |
| Phosphate (mmol/L) | 0.06 | 0.07 | -0.01( -0.19- 0.18) |
| Calcium (mmol/L) | 0.02 | 0.01 | 0.01( -0.11- 0.14) |
| Magnesium (mmol/L) | 0.01 | -0.03 | 0.04( -0.02- 0.10) |
