# Supplementary material for: Comparing mixed oil to soybean oil lipid emulsion in patients on home parenteral nutrition: a pilot prospective double-blind, crossover, randomized trial
Source: Pilot Feasibility Stud. 2023 Apr 20;9:64. doi: 10.1186/s40814-023-01295-1 (PMC10116729; doi:10.1186/s40814-023-01295-1)
Supplement: Supplementary file 2 — Additional file 2: Supplementary Material Figure 1. Interaction betweenTreatment (Soybean Oil/Mixed Oil) and the change in liver enzymes on the changein ratio w-6: w-3. Supplementary Material Figure 2. Interaction between Treatment (Soybean Oil/MixedOil) and the change in liver enzymes on the change inOmega 6. Supplementary Material Figure 3. Interaction between Treatment (Soybean Oil/MixedOil) and the change inliver enzymes on the change in Omega 3. Supplementary Material Figure 4. Interaction between Treatment (Soybean Oil/MixedOil) and the change in liver enzymes on the change inEPA. Supplementary Material Figure 5. Interaction between Treatment (Soybean Oil/MixedOil) and the change in liver enzymes on the change inDHA. [file 40814_2023_1295_MOESM2_ESM.zip › Supplementary Material Figure Captions.docx]

**Supplementary Material Figure 1.** Interaction between Treatment (Soybean Oil/Mixed Oil) and the change in liver enzymes on the change in ratio w-6: w-3.

**Supplementary Material Figure 2.** Interaction between Treatment (Soybean Oil/Mixed Oil) and the change in liver enzymes on the change in Omega 6.

**Supplementary Material Figure 3.** Interaction between Treatment (Soybean Oil/Mixed Oil) and the change in liver enzymes on the change in Omega 3.

**Supplementary Material Figure 4.** Interaction between Treatment (Soybean Oil/Mixed Oil) and the change in liver enzymes on the change in EPA.

**Supplementary Material Figure 5.** Interaction between Treatment (Soybean Oil/Mixed Oil) and the change in liver enzymes on the change in DHA.
